# Computationally efficient, exact, covariate-adjusted genetic principal component analysis by leveraging individual marker summary statistics from large biobanks

**Published:** 2020

**Authors:** Jack M. Wolf, Martha Barnard, Xueting Xia, Nathan Ryder, Jason Westra, Nathan Tintle

**Affiliations:** Department of Mathematics, Statistics, and Computer Science, St. Olaf College, Northfield, MN 55057, USA; Department of Mathematics, Statistics, and Computer Science, St. Olaf College, Northfield, MN 55057, USA; Department of Mathematics and Statistics, Texas Tech University, Lubbock, TX 79409, USA; Department of Statistics, Colorado State University, Fort Collins, CO 80523, USA; Department of Math, Computer Science, and Statistics, Dordt University, Sioux Center, IA 51250, USA; Department of Math, Computer Science, and Statistics, Dordt University, Sioux Center, IA 51250, USA

**Keywords:** privacy, biobank, genetics, genome-wide association study, meta-analysis, multivariate analysis, computational challenges, data security, phenotypes

## Abstract

The popularization of biobanks provides an unprecedented amount of genetic and phenotypic information that can be used to research the relationship between genetics and human health. Despite the opportunities these datasets provide, they also pose many problems associated with computational time and costs, data size and transfer, and privacy and security. The publishing of summary statistics from these biobanks, and the use of them in a variety of downstream statistical analyses, alleviates many of these logistical problems. However, major questions remain about how to use summary statistics in all but the simplest downstream applications. Here, we present a novel approach to utilize basic summary statistics (estimates from single marker regressions on single phenotypes) to evaluate more complex phenotypes using multivariate methods. In particular, we present a covariate-adjusted method for conducting principal component analysis (PCA) utilizing only biobank summary statistics. We validate exact formulas for this method, as well as provide a framework of estimation when specific summary statistics are not available, through simulation. We apply our method to a real data set of fatty acid and genomic data.

## 1. Introduction

The availability of large amounts of disease, environmental, and genomic data provide researchers with unprecedented opportunities to explore the effect of genetic variants on phenotypes related to human health and, consequently, change the way we think about and treat diseases. Of specific interest are complex diseases with widespread impacts on societal wellbeing and that have largely unique etiology for each individual (e.g., cardiovascular disease, cancer, mental health). The wealth of individual level data in biobanks presents the potential opportunity to characterize the genetic architecture of complex diseases that could, in turn, allow for the personalization of treatments. While this expanse of health and genetic information provides exciting possibilities, there are still many concerns associated with using this large amount of data.^1^ The size of these datasets presents issues with computation costs, processing time, and data sharing. The confidential nature of genetic and phenotypic data also raises concerns regarding data privacy and security while transporting and using the data.^2,3^

Currently, various organizations (such as GeneAtlas with the UK Biobank) publish summary statistics, such as results from simple linear regressions (e.g., effect size estimates and standard errors), between all combinations of phenotypes and genotypes in biobank data on hundreds of thousands of individuals.^4,5^ The use of these summary statistics alleviates many of the issues associated with privacy and security, as there is no individually identifiable information being shared. In addition, the use of summary statistics greatly diminishes the size of the analysis dataset, making the transport of data simpler and more efficient. Finally, the fact that the biobank runs these simple, but computationally intensive, analyses diminishes the computational cost and time of analyses for individual research groups.

While the use of summary statistics in downstream analyses alleviate many of the problems associated with the use of large datasets, they limit researchers in the complexity of the analysis they can run. Biobanks often provide summary statistics that describe the relationship between genotypes and a single, simple phenotype, but many researchers are interested in complex combinations of phenotypes that more accurately describe clinically or biologically relevant traits. These same issues arise in the performance of meta-analysis, since meta-analysis can only investigate phenotypes as complex as the summary statistics that each individual cohort provides. However, more complex phenotypes are important to explore in genome wide association studies (GWAS), as analyzing combinations of phenotypes can help explore various genetic mechanisms behind specific traits of interest, such as pleiotropy between correlated phenotypes.^6^ The flexibility to explore complex phenotypes is especially important in a meta-analysis, as the statistical power of the analysis of simple phenotypes might prompt unanticipated research questions. To continue to circumvent the computational and privacy problems in biobanks and meta-analyses and answer biologically relevant research questions, we need a way to explore complex traits (phenotypes) through these simple summary statistics.

There is limited knowledge of how we can use published summary statistics for these more complex analyses. Ultimately, we wish to know whether we can make inferences about the relationship between genotypes and the combined phenotype *y* = *f*(*y*_1_, *y*_2_, …, *y*_*m*_) if we know the relationships between the genotypes with the individual phenotypes *y*_1_, *y*_2_,…, *y*_*m*_. Recently Gasdaska et al. (2019) provided a method to summarize a regression of a linear combination of known phenotypes against genotypes, and other studies have provided new multivariate methods for exploring multiple phenotype associations with GWAS summary statistics.^7–11^ Others have explored how to investigate these multiple phenotype associations within the context of a meta-analysis through summary statistics.^12–14^ Furthermore, simple methods such as covariate adjustment and traditional multivariate methods can be used to explore multiple phenotype associations.^15^ Multivariate methods such as principal component analysis (PCA) have also been used in GWAS and meta-analysis to increase the power of the analysis, which allows for the exploration of rarer genetic variants.^16,17^

While these individual methods are mathematically intuitive or have the ability to explore correlated phenotypes, we have not found a method that focuses on doing both. Previous studies have provided various complicated, yet effective techniques, but these techniques cannot be intuitively applied to a wide variety of GWAS situations. Therefore, we bridge the gap between existing methods by providing a simple, mathematically intuitive method which allows the exploration of multiple phenotype associations than can be used in the context of both a single GWAS or a meta-analysis. We present a method that provides formulas for the slopes, intercept, and standard error for a PCA of phenotypes of interest, while allowing for a user-specified set of covariates utilizing only widely available biobank summary statistics. We will first demonstrate our method of covariate adjustment for any number of covariates and phenotypes, and then demonstrate a method for performing PCA with summary statistics. We will validate these methods through simulation as well as a real data application of our methods to fatty acid and genotype data from the Framingham Heart Study.

## 2. Methods

### 2.1. Notation

Throughout this paper, we use the matrix **Y** to denote an *n* × *m* matrix of observations of *m* phenotypes across *n* subjects. The column vector **y**_*h*_ represents *n* observations on the *h*th phenotype where *h* ∈ {1, 2, …, *m*}. That is, **y**_*h*_ = [*y*_*h*1_ · · ·*y*_*hn*_]′. Similarly, we will use the matrix **X** to denote an *n* × (*p* + 1) design matrix of *n* observations on *p* covariates, for *p >* 1. We will use the matrix **X**_*k*_ to reference a *n* × 2 design matrix with only 1 covariate, **x**_*k*_, for any *k* ∈ {1, 2, *…, p*}. For each simple linear regression model fit for **y**_*h*_ ~ **x**_*k*_, we use the notation **y**_*h*_ = **X**_*k*_*β*_*hk*_, where *β*_*hk*_ is a 2 × 1 vector of model coefficients. We will use *b*_*hk*_ to reference the “slope” coefficient, or the second element of the vector *β*_*hk*_. For each multiple linear regression model fit for **y**_**h**_ ~ **X** we use the notation **y**_*h*_ = **X***β*_*h*_, where *β*_*h*_ is a (*p* + 1) × 1 vector of model coefficients.

We will frequently use the following formulas in the paper. For any response vector **y** where **y** = **X***β* + *ε*:

(1) $$\beta ={\left(X^{\prime }X\right)}^{-1}X^{\prime }y$$

(2) $$\mathrm{var}(\beta )={\hat{\sigma }}^{2}{\left(X^{\prime }X\right)}^{-1},$$

where ${\hat{\sigma }}^{2}$ is isthe sum of squared residuals divided by degrees of freedom.

### 2.2. Assumptions

We assume we have the following summary statistics: slope and intercept estimates for simple linear regressions of each phenotype as a function of the genotype, minor allele frequency and variance of the genotypes (which can be estimated via minor allele frequency if necessary), and covariance matrix of the phenotypes. While having a known covariance matrix of the phenotypes makes the following methods exact calculations, we will also demonstrate the accuracy of our methods using the following estimation used in Gasdaska et al. (2019)^7^ and similar to those proposed in Zhu et al. (2015)^14^ and Kim et al. (2015).^18^ For *h, j* ∈ {1, 2, …, *m*},

(3) $$\mathrm{cov}\left(y_{h},\;y_{j}\right)=\mathrm{cor}\left(y_{h},\;y_{j}\right)\sqrt{\mathrm{var}\left(y_{h}\right)\mathrm{var}\left(y_{j}\right)}\approx \mathrm{cor}\left(b_{h},\;b_{j}\right)\sqrt{\mathrm{var}\left(y_{h}\right)\mathrm{var}\left(y_{j}\right)},$$

where **b**_*h*_ and **b**_*j*_ are vectors of slope coefficients from simple linear regressions of **y**_*h*_ against every genotype, and **y**_*j*_ against every genotype, respectively.

### 2.3. Covariate Adjustment

#### 2.3.1. Single Phenotype

Suppose that we have fit models for **y**_*h*_ ~ **x**_1_, **y**_*h*_ ~ **x**_2_, …, **y**_*h*_ ~ **x**_*p*_ and wish to describe the linear model **y**_*h*_ ~ **X**, or **y**_*h*_ = **X***β* + *ε*.

To solve for *β*, we turn to Equation 1. Now,

(4) $$X^{\prime }X=\left[\begin{array}{cccc} n & \sum_{i=1}^{n}x_{1i} & \cdots & \sum_{i=1}^{n}x_{pi}\\ \sum_{i=1}^{n}x_{1i} & \sum_{i=1}^{n}x_{1i}^{2} & \cdots & \sum_{i=1}^{n}x_{1}x_{pi}\\ \vdots & \vdots & \ddots & \vdots \\ \sum_{i=1}^{n}x_{pi} & \sum_{i=1}^{n}x_{p}x_{1i} & \cdots & \sum_{i=1}^{n}x_{pi}^{2} \end{array}\right],$$

Where

$$\begin{array}{l} \;\;\;\;\;\sum_{i=1}^{n}x_{ki}={\bar{x}}_{k}n,\\ \sum_{i=1}^{n}x_{ki}x_{li}=\mathrm{cov}\left({\text{x}}_{k},{\text{x}}_{l}\right)(n-1)+{\bar{\text{x}}}_{k}{\bar{\text{x}}}_{l}n \end{array}$$

for any *k, l* ∈ {1, 2, …, *p*}. For a single phenotype multiplied by a constant *c*_*h*_, *c*_*h*_**y**_*h*_,

(5) $$X^{\prime }c_{h}y_{h}=c_{h}\left[\begin{array}{c} \sum_{i=1}^{n}y_{hi}\\ \sum_{i=1}^{n}x_{1i}y_{hi}\\ \vdots \\ \sum_{i=1}^{n}x_{pi}y_{hi} \end{array}\right],$$

Where

$$\begin{array}{l} \;\;\;\;\;\sum_{i=1}^{n}y_{hi}={\bar{y}}_{h}n,\\ \sum_{i=1}^{n}x_{ki}y_{hi}={\hat{b}}_{hk}\mathrm{var}\left({\text{x}}_{k}\right)(n-1)+{\bar{\text{x}}}_{k}{\bar{\text{y}}}_{h}n. \end{array}$$

To calculate *β* we solve for these matrices and apply them to Equation 1.

We can manipulate Equation 2 to solve for the standard error of our coefficients. By substitution, we have:

(6) $$\mathrm{var}(\beta )={\hat{\sigma }}^{2}{\left(X^{\prime }X\right)}^{-1}=\frac{c_{h}^{2}y_{h}^{\prime }y_{h}-\beta^{\prime }X^{\prime }c_{h}y_{h}}{n-(p+1)}{\left(X^{\prime }X\right)}^{-1}.$$

To compute this matrix we use our calculated $\hat{\beta }$, $X^{\prime }X$, and $X^{\prime }c_{h}y_{h}$. Then,

$$c_{h}^{2}{\text{y}}_{h}^{\prime }{\text{y}}_{h}=c_{h}^{2}\sum_{i=1}^{n}y_{hi}^{2}=c_{h}^{2}\left(\mathrm{var}\left({\text{y}}_{h}\right)(n-1)+{\bar{\text{y}}}_{h}^{2}n\right).$$

Using these matrices we can compute the matrix var $\left(\hat{\beta }\right)$. To calculate SE $\left({\hat{\beta }}_{j}\right)$ we take the square root of the *j*th diagonal entry of var $\left(\hat{\beta }\right)$

#### 2.3.2. Linear Combination of Phenotypes

Suppose we want to analyze a linear combination of all phenotypes in the matrix **Y** while adjusting for covariates.

We still will use Equation 1 to calculate our slope vector. *β*. To do so, we can still calculate **X′X** through Equation 4. However, to calculate **X′y** for a linear combination of phenotypes $\text{y}=c_{1}{\text{y}}_{1}+c_{2}{\text{y}}_{2}+\cdots +c_{m}{\text{y}}_{\text{m}}$,

(7) $$X^{\prime }y=\left[\begin{array}{c} c_{1}\sum_{i=1}^{n}y_{1i}+c_{2}\sum_{i=1}^{n}y_{2i}+\cdots +c_{m}\sum_{i=1}^{n}y_{mi}\\ c_{1}\sum_{i=1}^{n}x_{1i}y_{1i}+c_{2}\sum_{i=1}^{n}x_{1i}y_{2i}+\cdots +c_{m}\sum_{i=1}^{n}x_{1i}y_{mi}\\ \vdots \\ c_{1}\sum_{i=1}^{n}x_{pi}y_{1i}+c_{2}\sum_{i=1}^{n}x_{pi}y_{2i}+\cdots +c_{m}\sum_{i=1}^{n}x_{pi}y_{mi} \end{array}\right],$$

Where

$$\begin{array}{l} \;\;\;\;\;\;\;\;\;\;\;\;\;c_{1}\sum_{i=1}^{n}y_{1i}+c_{2}\sum_{i=1}^{n}y_{2i}+\cdots +c_{m}\sum_{i=1}^{n}y_{mi}=n\left(c_{1}{\bar{y}}_{1}+c_{2}{\bar{y}}_{2}+\cdots +c_{m}{\bar{y}}_{m}\right),\\ c_{1}\sum_{i=1}^{n}x_{k}y_{1i}+c_{2}\sum_{i=1}^{n}x_{k}y_{2i}+\cdots +c_{m}\sum_{i=1}^{n}x_{k}y_{mi}=\left(c_{1}{\hat{b}}_{1k}+c_{2}{\hat{b}}_{1k}+\cdots +c_{m}{\hat{b}}_{mk}\right)\mathrm{var}\left({\text{x}}_{k}\right)(n-1)\\ \;+n{\bar{x}}_{k}\left(c_{1}{\bar{y}}_{1}+c_{2}{\bar{y}}_{2}+\cdots +c_{m}{\bar{y}}_{m}\right). \end{array}$$

Note that if we already have summary statistics for covariate-adjusted models (${\hat{\beta }}_{1},{\hat{\beta }}_{2},\ldots ,{\hat{\beta }}_{m}$ for ${\text{y}}_{1}\sim \text{X},{\text{y}}_{2}\sim \text{X},\ldots ,{\text{y}}_{\text{m}}\sim \text{X}$), Equation 1 simplifies to the following:

(8) $$\hat{\beta }=c_{1}{\hat{\beta }}_{1}+c_{2}{\hat{\beta }}_{2}+\cdots +c_{m}{\hat{\beta }}_{m}.$$

To calculate standard errors for this linear combination, we have

(9) $$\mathrm{var}(\beta )=\frac{{\text{y}}^{\prime }\text{y}-\beta^{\prime }{\text{X}}^{\prime }\text{y}}{n-(p+1)}{\left({\text{X}}^{\prime }\text{X}\right)}^{-1}.$$

We can then evaluate Equation 9 using **Xy** calculated from Equation 7, *β* calculated from Equation 1, and

(10) $$y^{\prime }y=\sum_{h=1}^{m}\sum_{j=1}^{m}c_{h}c_{j}\left(\mathrm{cov}\left(y_{h},y_{j}\right)(n-1)+{\bar{y}}_{h}{\bar{y}}_{j}n\right)$$

for *h, j* ∈ {1,2, …, *m*}.

### 2.4. Principal Component Analysis

Assume that **Y** is centered. That is, that ${\bar{y}}_{h}=0$ for all *h* ∈ {1, 2, …, *m*}. Then, if *λ*_*j*_ is the *j*th highest eigen-value of cov(**Y**), with associated eigen-vector ${\left[\phi_{j1}\cdots \phi_{jh}\right]}^{\prime }$ it follows that $\phi_{j1}y_{1}+\cdots +\phi_{jh}y_{h}$ is the *j*th principal component score of **Y**. So, the previously discussed methods can be applied to calculate the coefficients and standard errors of the model

$$\phi_{jh}y_{1}+\cdots +\phi_{jh}y_{h}=X\beta +\varepsilon .$$

#### 2.4.1. Standardizing and Centering

If the summary statistics do not center **Y**, we can post-hoc transform the summary statistics to center **Y** (and optionally standardize **Y**). If **y**_*h*_ has mean *μ*_*h*_, standard deviation *σ*_*h*_, and ***y**_h_* = **X***β*_*h*_+*ε*_*h*_, then regression coefficients describing a centered **y**_*h*_ with the same covariates can be found by subtracting *μ*_*h*_ from the intercept and leaving all other coefficients unchanged. Standard errors remain unchanged with centering. Further, if we wish to standardize **y**_*h*_, regression coefficients can be found by subtracting *μ*_*h*_ from the intercept, and then diving all coefficients by *σ*. Standard errors for the standardized response’s coefficients are equivalent to their unstandardized standard errors divided by $\sigma_{h}^{2}$.

### 2.5. Simulation

We simulated genomes across 2,000 subjects 1,000 times. Each genome consisted of 100,000 SNPs with minor allele frequencies generated from a beta distribution. Each subject had 5 phenotypes: age, sex, **y**_1_, **y**_2_, and **y**_3_. Subjects’ ages and sexes were generated from Poisson and Bernoulli distributions, respectively. We generated our primary response phenotypes (**y**_1_, **y**_2_, and **y**_3_) to be associated with the first 10 SNPs, age, and sex. As a result of this specification, we saw average correlations of 0.30 between **y**_1_ and **y**_2_, −0.08 between **y**_1_ and **y**_3_, and 0.07 between **y**_2_ and **y**_3_ across all simulations.

#### 2.5.1. Post-Hoc Covariate Adjustment Simulation

To address our post-hoc covariate adjustment, we first calculated slope coefficients and standard errors for the regression **y**_**1**_ ~ SNP + age + sex and compared them to these values calculated using our methods with simple linear regression summary statistics. We calculated these values both using the true covariance matrix of our phenotypes, and using Equation 3 to approximate the phenotype covariance matrix.

#### 2.5.2. Principal Component Analysis Simulation

To address our PCA method, we calculated the principal component weights on **y**_1_, **y**_2_, and **y**_3_ and calculated slope coefficients and standard errors for the regression of the first principal component against SNP, age, and sex. We compared these values to those calculated using our methods with known summary statistics of **y**_*h*_ ~ SNP + age + sex for *h* ∈ {1, 2, 3}. We calculated these values both using the true covariance matrix of our phenotypes, and using Equation 3 to approximate the phenotype covariance matrix.

### 2.6. Real Data Example

Previous genome wide association studies explored associations between SNPs and red blood cell fatty acid (RBC FA) levels indicative of various health measures such as cardiovascular health and inflammation using data from The Framingham Heart Study.^19–21^ We applied our method to unrelated individuals in the Generation 3 and Offspring cohorts with a sample size of 1,454 with data on 408,595 SNPs after quality control. We investigated the Omega-3 and Omega-6 fatty acids. The production of Omega-3s and Omega-6s are highly related and therefore it is useful to determine how genotypes are associated with each of these groups, rather than each fatty acid individually. We did this by performing regressions on the principal components of the 4 Omega-3 and the 3 Omega-6 fatty acids. We performed both our posthoc covariate adjustment and PCA methods on the summary statistics of single marker tests for each fatty acid and covariate, and compared the results to models run in the traditional framework. We ran the models with two different sets of covariates: one set included the covariates age, sex, and cohort, while the other also included the other fatty acid group as covariates. Look to cited studies for more information regarding the results of past fatty acid GWAS and the Framingham cohort.^19–21^

## 3. Results

### 3.1. Simulation Results

#### 3.1.1. Post-Hoc Covariates Adjustment

Our method to describe covariate adjusted models proved to be exact to rounding errors when we assumed the true phenotype covariance matrix. We had mean slope error −1.68 × 10^−18^ with mean intra-genomic variance 3.78 × 10^−33^ (max intra-genomic variance 1.52 × 10^−32^). Our standard error estimate had mean error 1.67 × 10^−20^ with mean intra-genomic variance 9.01 × 10^−33^ (max intra-genomic variance 5.62 × 10^−32^).

When estimating the phenotype covariance matrix, our approximation still performed well. Our estimate of the slope had mean error 1.87 × 10^−9^ with mean intra-genomic variance 2.99 × 10^−9^ (max intra-genomic variance 4.12 × 10^−8^). The standard error estimate had mean error 5.25 × 10^−8^ and mean intra-genomic variance 7.77 × 10^−13^ (max intra-genomic variance 1.96 × 10^−11^).

#### 3.1.2. Principal Component Analysis

Our method to describe models that incorporated principal components proved to be exact to rounding errors when we assumed the true phenotype covariance matrix. Our slope estimate had mean error −2.48×10^−19^ with mean intra-genomic variance 2.64×10^−33^ (max intra-genomic variance 3.25 × 10^−32^). Our slope standard error estimate had mean error −3.30 × 10^−19^ with mean intra-genomic variance 5.66 × 10^−35^ (max intra-genomic variance (2.84 × 10^−34^).

**Fig. 1:**
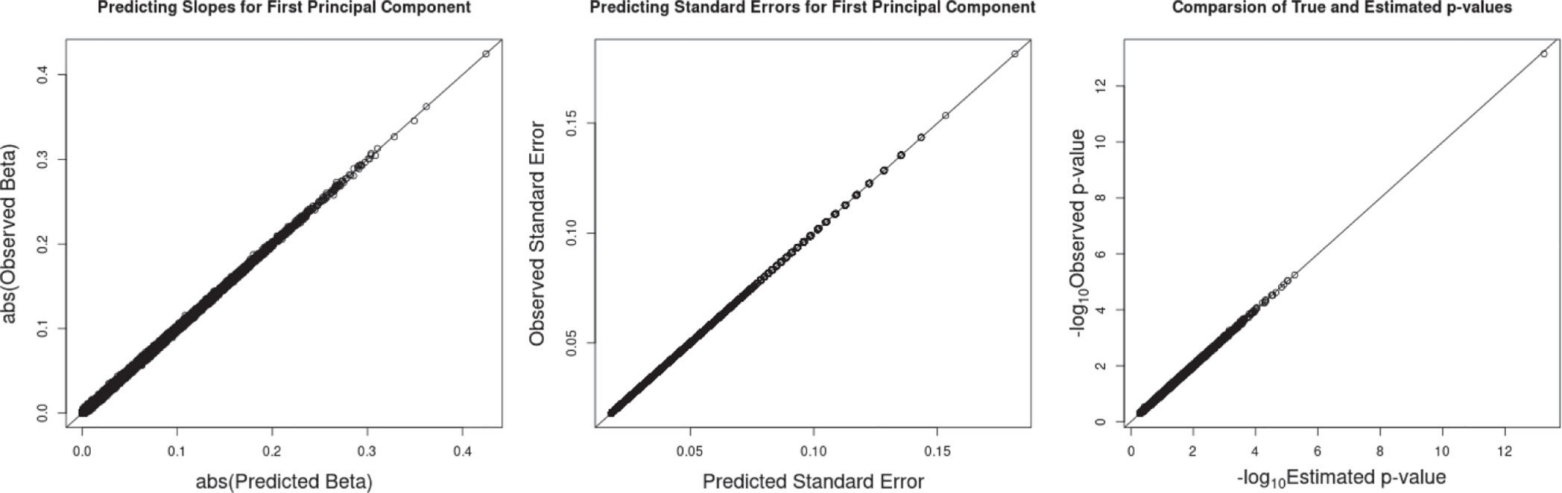
Differences of our method’s approximations of slope, standard error of slope, and *p-*values and those achieved when fitting a model for the first principal component on the raw data. These figures illustrate the high accuracy of our method, even when approximating the covariance structure of the phenotypes. (a) Difference of observed and predicted SNP slope coefficients on simulated data when approximating phenotype covariance. (b) Difference of observed and predicted standard errors of the SNP slope coefficient on simulated data when approximating phenotype covariance. (c) Difference of observed and predicted p-values of SNPs and the first principal component on simulated data when approximating phenotype covariance.(−log_10_ scale)

When approximating the covariance of y_1_, y_2_, and y_3_, our estimate still performed well. Across all 1,000 genotypes, our slope estimate had a mean error of 2.00 × 10^−7^ with mean intra-genomic variance 5.11 × 10^−7^ (max intra-genomic variance 1.75 × 10^−5^). Our standard error estimate had a mean error of 8.85 × 10^−7^ with mean intra-genomic variance 2.70 × 10^−10^ (max intra-genomic variance 7.91 × 10^−9^). Figure 1 displays the accuracy of our method on the first simulated genome.

### 3.2. Real Data Example Results

#### 3.2.1. Method Accuracy

**Fig. 2:**
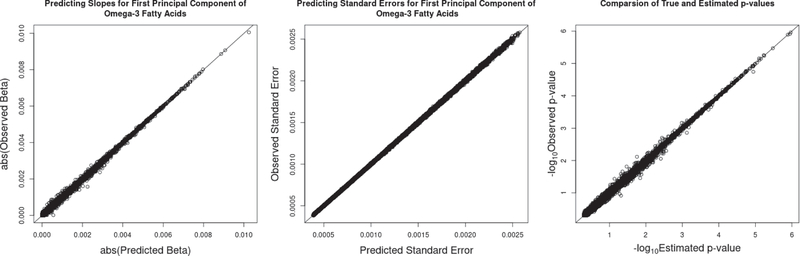
Differences of our method’s approximation of SNP slope coefficients, slope standard errors, and *p*-values on the first principal component of Omega-3 fatty acids, adjusting for age, sex, and cohort using data from the Framingham Heart Study. These figures show our method’s high accuracy. (a) Approximated and true slopes of the first principal component of Omega-3 fatty acids on FHS data. (b) Approximated and true slope standard errors of the slope of the first principal component of Omega-3 fatty acids on FHS data. (c) Difference in observed and predicted p-values of the first principal component of Omega-3 fatty acids on FHS data.(−log_10_ scale)

Our method approximated the results of models fit on raw subject-level data with high accuracy and low variance. Table 1 displays our method’s accuracy for all responses with and without adjustment for fatty acid covariates. These models show more variation than in simulation due to deviations from Hardy-Weinberg equilibrium (HWE) and missing data that affected values such as the means of the phenotypes. At a significance threshold of 2 × 10^−7^, our method reached the same conclusions as models fit on the raw data for every SNP. We display the accuracy of our model for the first principal component of Omega-3 fatty acids, adjusting for age, sex, and cohort in Figure 2.

#### 3.2.2. Analysis of Hits

The post-hoc covariate adjustment on both individual fatty acids and PCA for the Omega-3 and Omega-6 fatty acids hit genes that have been found in previous GWAS on fatty acids such as FADS1, ELOVL2, and LPCAT3.^19–21^ Using principal components and covariate adjustment we found a novel gene that has not yet been found associated with fatty acids before: PTPRM, and another (AGPAT4) that was only identified with a fatty acid ratio before on this sample.^19^ Table 2 displays all SNPs found significant with any individual Omega-3 or Omega-6 fatty acid, or the first, second, or third principal components of either Omega-3 or Omega-6 fatty acids.

## 4. Discussion

We have developed exact methods for describing the relationship between phenotypes and genotypes for covariate adjusted linear combinations of any number of phenotypes (including post-hoc covariate adjustment) as well as for PCA using summary statistics. We have supplied the mathematical frameworks for these methods and validated them through a simulation and a real data example of both post-hoc covariate analysis and PCA, as well as the combination of the two.

We have provided a simple, efficient method for utilizing covariates and PCA in GWAS and GWAS meta-analyses using only summary statistics. In a GWAS, these methods save in computation time, and cost, as well as the time and size of data transfers. The post-hoc covariate adjustment also allows researchers to explore multiple phenotype associations through adding phenotypes correlated with the response phenotype as covariates in a computationally and time efficient way. The use of our covariate and PCA method becomes even more time-saving in a meta-analysis, as individual cohorts do not need to rerun and resend more complex analyses for the meta-analysis in order to explore more complex phenotypes or covariate adjustments. The PCA method can also be applied to a principal component meta-analysis by using methods from Ried et al. (2016) to compute universal weights that are applied to individual cohort summary statistics.^17^ Our real data application also demonstrates that covariate adjustment and PCA can and do affect the SNPs found in GWAS results and thus might lead to the exploration of new gene associations, and identified a novel gene.

Even though our method is a useful tool to flexibly explore biologically meaningful phenotypes, we suggest that future work continue to explore leveraging summary statistics to explain other complex phenotypes. For example, multiplied phenotypes can explain both logical and and or statements as: “*y*_1_ and *y*_2_” = *y*_1_·*y*_2_ and “*y*_1_ or *y*_2_” = *y*_1_+*y*_2_−*y*_1_·*y*_2_. These logical statements help describe how many diseases are clinically diagnosed, and thus would aid in explaining the relationship between genetics and these diseases. Future work can also explore how to expand these methods into linear mixed-effects models in order to incorporate kinship matrices and account for relatedness in these models. We are also currently working on an R package that will perform the calculations for these methods to help their implementation.

We also must acknowledgement some limitations of our method. Throughout our mathematical framework we assume that the genotypes follow HWE. Assuming HWE means that knowing the minor allele frequency of a genotype gives exact calculations for values such as the mean and variance of the genotype. In practice, not all genotypes included in a GWAS analysis exactly follow HWE, and thus future work should explore the robustness of this in assumption in practice, though we anticipate minimal impact in downstream analysis. Our real data analysis shows a representative application of the method; however, future work should continue to explore practical issues involved in the implementation of the method on real data. Detailed results not shown demonstrate that this method is minimally impacted by non-differential genotype errors in biobanks.

Use of summary statistics to share both biobank data and individual cohort analyses within a meta-analysis alleviate many issues with privacy, data size and transfer, as well as computational cost and time, while the data itself presents an unprecedented opportunity to explore human health and genetically complex phenotypes. Our method provides exact formulas along with estimation techniques for using these summary statistics for covariate-adjusted linear models and multivariate methods, that in turn can help explain the biological mechanisms between phenotypes of interest. We have continued the work of previous methodological advances by leveraging these summary statistics to investigate the relationship between genetics and diseases. Future work will explore additional methods of combining phenotypes.

## Supplementary Material

1

## Figures and Tables

**Table 1:** The accuracy of our method to estimate the first and second principal components of Omega-3 and Omega-6 fatty acids. Errors were minimal with low variance in all cases. A portion of these errors can be explained by deviations from HWE and missing genotype data.

Response	Adjustments	Mean Slope Error	Mean % Slope Error	Variance Slope Error	Mean SE Error	Variance SE Error

Omega-3, PC1	Age, Sex, Cohort	1.03 × 10^−7^	2%	9.19 × 10^−11^	−1.57 × 10^−7^	3.66 × 10^−12^
Omega-3, PC2	Age, Sex, Cohort	−1.67 × 10^−8^	2%	1.13 × 10^−11^	2.04 × 10^−9^	4.34 × 10^−13^
Omega-3, PC1	Age, Sex, Cohort, Omega-6 FA	4.95 × 10^−8^	4%	6.53 × 10^−11^	1.17 × 10^−8^	2.42 × 10^−11^
Omega-3, PC2	Age, Sex, Cohort, Omega-6 FA	−1.45 × 10^−8^	4%	1.27 × 10^−11^	2.50 × 10^−8^	4.14 × 10^−13^
Omega-6, PC1	Age, Sex, Cohort	1.71 × 10^−7^	3%	2.82 × 10^−10^	2.04 × 10^−8^	1.86 × 10^−11^
Omega-6, PC2	Age, Sex, Cohort	4.88 × 10^−8^	2%	8.07 × 10^−11^	−8.72 × 10^−8^	4.17 × 10^−12^
Omega-6, PC1	Age, Sex, Cohort, Omega-3 FA	9.96 × 10^−8^	2%	2.59 × 10^−10^	−2.18 × 10^−8^	8.64 × 10^−12^
Omega-6, PC2	Age, Sex, Cohort, Omega-3 FA	5.27 × 10^−8^	3%	7.98 × 10^−11^	−4.07 × 10^−8^	3.11 × 10^−12^

**Table 2:** Results of significant (*p* < 2 × 10^−7^) SNPs from Fatty Acids comparing models with and without fatty acids as covariates. Our method and traditional methods on the raw data found the same SNPs significant in all cases.

# of SNPs	Chr	Pos	Gene	Significant w/ out FA Covariates	Significant w/ FA Covariates

11	6	10954307–11050290	ELOVL2	DPA, O3PC2	O3PC2, O3PC1
1	6	161187057	AGPAT4		O6PC3
10	11	61781986–61888710	FADS1	LA, ADA, Adrenic, O6PC1, O6PC2	O6PC1, O6PC2, O3PC1, O3PC3
5	12	6966719–7013532	LPCAT3	LA, O6PC1	O6PC1, O3PC1
2	12	7057810–7069674	None	LA, O6PC1	
1	18	7881144	PTPRM	O3PC3	
