# Supplementary material for: Computationally efficient, exact, covariate-adjusted genetic principal component analysis by leveraging individual marker summary statistics from large biobanks
Source: Pac Symp Biocomput. Author manuscript; Available in PMC 2020 Jan 1. (PMC6907735)
Supplement: 1 [file NIHMS1061512-supplement-1.pdf]

Supplementary Materials for ***Computationally efficient, exact covariate-adjusted multivariate methods for genetic analysis leveraging summary statistics from large biobanks***

Theorem 1 (Equation 8). *Let* $\mathbf{y_1} = \mathbf{X}\beta_1 + \varepsilon_1$, $\mathbf{y_2} = \mathbf{X}\beta_2 + \varepsilon_2, \ldots, \mathbf{y_m} = \mathbf{X}\beta_m + \varepsilon_m$. *Then, for* $c_1\mathbf{y_1} + c_2\mathbf{y_2} + \cdots + c_m\mathbf{y_m} = \mathbf{X}\beta$,

$$\widehat{\beta} = c_1\widehat{\beta}_1 + c_2\widehat{\beta}_2 + \cdots + c_m\widehat{\beta}_m.$$

Proof. Define $\mathbf{y} = c_1\mathbf{y_1} + c_2\mathbf{y_2} + \cdots + c_m\mathbf{y_m}$. Then,

$$\begin{aligned}
c_1\mathbf{X}'\mathbf{y_1} + c_2\mathbf{X}'\mathbf{y_2} + \cdots + c_m\mathbf{X}'\mathbf{y_m} &= c_1 \begin{bmatrix} \sum_{i=1}^n y_{1i} \\ \sum_{i=1}^n x_{1i}y_{1i} \\ \vdots \\ \sum_{i=1}^n x_{pi}y_{1i} \end{bmatrix} + c_2 \begin{bmatrix} \sum_{i=1}^n y_{2i} \\ \sum_{i=1}^n x_{1i}y_{2i} \\ \vdots \\ \sum_{i=1}^n x_{pi}y_{2i} \end{bmatrix} + \cdots + c_m \begin{bmatrix} \sum_{i=1}^n y_{mi} \\ \sum_{i=1}^n x_{1i}y_{mi} \\ \vdots \\ \sum_{i=1}^n x_{pi}y_{mi} \end{bmatrix} \\
&= \begin{bmatrix} c_1\sum_{i=1}^n y_{1i} + c_2\sum_{i=1}^n y_{2i} + \cdots + c_m\sum_{i=1}^n y_{mi} \\ c_1\sum_{i=1}^n x_{1i}y_{1i} + c_2\sum_{i=1}^n x_{1i}y_{2i} + \cdots + c_m\sum_{i=1}^n x_{1i}y_{mi} \\ \vdots \\ c_1\sum_{i=1}^n x_{pi}y_{1i} + c_2\sum_{i=1}^n x_{pi}y_{2i} + \cdots + c_m\sum_{i=1}^n x_{pi}y_{mi} \end{bmatrix} \\
&= \mathbf{X}'\mathbf{y}.
\end{aligned}$$

Therefore,

$$\begin{aligned}
\beta &= (\mathbf{X}'\mathbf{X})^{-1}\mathbf{X}'\mathbf{y} \\
&= (\mathbf{X}'\mathbf{X})^{-1}(c_1\mathbf{X}'\mathbf{y_1} + c_2\mathbf{X}'\mathbf{y_2} + \cdots + c_m\mathbf{X}'\mathbf{y_m}) \\
&= c_1(\mathbf{X}'\mathbf{X})^{-1}\mathbf{X}'\mathbf{y_1} + c_2(\mathbf{X}'\mathbf{X})^{-1}\mathbf{X}'\mathbf{y_2} + \cdots + c_m(\mathbf{X}'\mathbf{X})^{-1}\mathbf{X}'\mathbf{y_m} \\
&= c_1\widehat{\beta}_1 + c_2\widehat{\beta}_2 + \cdots + c_m\widehat{\beta}_m,
\end{aligned}$$

as desired. □
